# Could stress granules be involved in age-related diseases?

**DOI:** 10.18632/aging.100090

**Published:** 2009-09-21

**Authors:** Imed-Eddine Gallouzi

**Affiliations:** McGill University, Department of Biochemistry, Rosalind and Morris Goodman Cancer Center, Montreal, Quebec, Canada

**Keywords:** senescence, stress granules, oxidative stress

Cellular senescence, an irreversible cell-cycle arrest, acts as a safeguard program that limits the proliferative capacity of cells when exposed to endogenous or exogenous stress signals [1,2]. However, the senescence phenotype is also considered as a sign that the life span of a cell has reached its end. Indeed, in the early 1960s, Hayflick and Moorhead showed that despite the maintenance of optimal culturing conditions for a long period of time, normal cells do not proliferate forever [3-5]. For example, they observed that normal human diploid fibroblasts have a limited proliferation capacity and after a finite number of population doubling, they stop dividing and enter senescence. The occurrence of replicative senescence has been demonstrated for most cell types, with a few relevant exceptions including embryonic germ cells [6]. In human cells, the primary cause of cellular senescence appears to be the progressive shortening of telomeres, which are DNA structures at the end of eukaryotic chromosomes [7-9]. Senescence can also be induced by non-telomeric signals, termed "premature" or "accelerated" senescence [10]. Senescence-inducing signals, such as DNA-damage response (DDR) and oxidative stress (OS), usually engage either the p53 or the cyclin-dependent kinase inhibitor p16 pathway [11-14]. Active p53 establishes senescence, in part, by inducing the expression of the cyclin-dependent kinase inhibitor p21^cip^, which suppresses the phosphorylation of the retinoblastoma protein pRB, leading to its inactivation [15].

The importance of the senescence phenotype is underscored by the fact that this condition could trigger  two opposite outcomes. Due to its antiproliferative effect, senescence is activated to prevent further growth of transformed or sick cells that are subsequently eliminated by the immune system [10,16]. This effect has mainly been observed in young organisms, and as such senescence is considered a natural tumor suppressor mechanism [16-19]. On the other hand, the situation seems to change in aged individuals. Indeed, several studies have suggested that as we age, many senescent cells escape the immune system and end up accumulating in different tissues for a long period of time, which correlates with age-related diseases such as cancer [1,16,20]. This functional dichotomy of the senescence phenotype raises questions such as how and why the same conditions could lead to opposite outcomes depending on age. While the answers to these questions are still elusive, it is possible that the switch of senescent cells from being a natural break of tumor growth to becoming promoters of malignancy occurs over a long period of time as a consequence of repeated exposure to stress during the life-span of an organism. Since senescent cells remain metabolically active [7,19], these stresses could cause dramatic changes in the expression pattern of key genes which could explain how and why as we age senescent cells switch their function to become promoters of tumor growth. Although the expression patterns and the activities of the genes involved in promoting and maintaining the senescence state are well studied, very little is known about the effect stress could have on their expression when the cell are fully senescent.

To address the questions asked above we assessed how senescent cells, incapable of further division, react to extracellular assaults. Our recent work [21] clearly indicated that in response to stress, senescent cells activate mechanisms that are similar to those seen in exponentially growing cells. We observed that fully senescent cells exposed to stresses such as oxidative stress (OS) or heat shock are able to form *bonafide* stress granules (SGs) [21]. Originally, SGs were identified as cytoplasmic RNA granules that form in mammalian cells upon exposure to various stresses [22-24]. SG assembly represents one of the main prosurvival mechanisms through which cells cope with environmental assaults by helping them reprogram mRNA metabolism and repair stress-induced damage. We observed that when fully senescent human fibrobalsts exposed to either heat shock, or to arsenate (AS), a well-known inducer of OS, a much higher number of SGs form than in exponentially growing cells [21]. Since it is known that senescent cells have a decreased capacity to adapt to environmental stresses [25,26], we assessed the impact of this high number of SGs on their ability to recover from these assaults. We observed that upon switching cells to AS-free media, SG disassembly in fully senescent cells occurs at a slower rate than in cells at earlier stages of the senescence process. Recent experiments performed in my laboratory support this observation and showed that in fully senescent cells there is a small but reproducible decrease in the expression levels of the heat shock protein 70 (HSP70), which is one of the key players involved in cell recovery from a variety of stresses [27,28] (Figure 1). This already suggested that while senescent cells maintained the ability to form SGs in response to stress, their recovery process was affected. This result is consistent with previous *in vitro* and *in vivo* studies showing that the expression of HSP70 decreases in senescent cells exposed to stresses such as heat shock [29,30]. Hence, together these data argue that the slow recovery rate observed in fully senescent cells [21] could be explained by the delayed expression of HSP70 protein. While SGs are entities used as a protective mechanism under stressful conditions, the fact that they take more time to disassemble in senescent cells upon stress removal could indicate a delay in the synthesis of many vital proteins needed for the maintenance of the senescence status.

It is well accepted that in exponentially growing cells SGs recruit a variety of mRNA not only for protection from decay and sorting purposes but also to block their translation during cell exposure to stress [32]. As soon as the stress is relieved, however, SG disassemble and translation resumes. Hence, we investigated whether this could also be the case in senescent cells.  Our data  indicate that the effect of OS-induced SGs on the expression of the p21^cip^ mRNAs is dependent on the senescence stage of the cell [21]. We showed that although the p21^cip^ mRNAs colocalizes with SGs in both early and fully senescent cells, the synthesis of p21^cip^ protein was rapidly shut off only in fully senescent cells. The fact that these cells were treated with a sub-lethal dose of AS for only a short period of time (30 min), indicates that the events leading to the translation inhibition of p21^cip^ mRNA are triggered quickly and correlate with the assembly of SGs. This, however, does not explain why p21^cip^ translation is not affected in cells at earlier stages of the senescence process despite the fact that in these cells the p21^cip^ message is also rapidly recruited to SGs. Surprisingly, our data raise the possibility that senescent and normally growing cells use different molecular mechanism to assemble SGs in response to OS. We observed that in both early and fully senescent cells the phosphorylation of eIF2α, a key factor in AS-induced SG formation [33], is significantly reduced [21]. This result suggests that during the senescence process OS-induced SG formation switches from an eIF2α phosphorylation-dependent mechanism to a process that is independent of this posttranslational modification. Work from several groups including ours, have demonstrated the existence of an eIF2α phosphorylation-independent mechanism for SG assembly. Indeed, cells exposed to Pateamine A or hippuristanol, two well-known inhibitors of the eukaryotic translation initiation factor A (eIF4A) form SGs in an eIF2α phosphorylation-independent manner [34,35]. Although this or similar mechanisms could explain SGs formation in senescent cells exposed to OS, this possibility needs to be tested experimentally. Defining the mechanisms by which SGs assemble in senescent cells could open the door to screen for chemical inhibitors/activators that modulate SG formation in these cells. This could in turn provide tools to design new strategies that prevent senescent cell from promoting malignancy.

**Figure 1.**
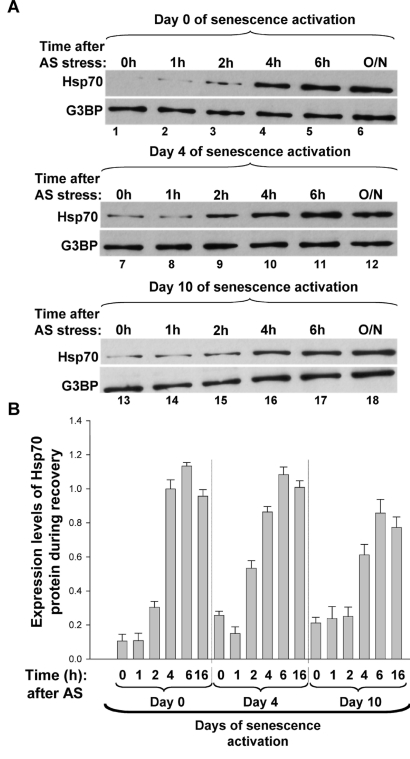
**The upregulation of Hsp70 expression correlates with SG disassembly during the recovery from AS stress of proliferative and senescent cells**. **(A-B)** Expression levels of Hsp70 protein after the removal of AS stress. (**A**) Proliferative and senescent IDH4 cells were incubated with arsenite (0.5mM) for 30 min. Cells were subsequently washed twice with PBS, replenished with fresh media and incubated for various periods of time at 37^o^C. Total cell extracts prepared from these cells were then used for Western blots analysis with antibodies specific to Hsp70 and G3BP  (used as the loading control). Representative western blots of three independent experiments are shown. (**B)** The bar graphs represent the expression level of Hsp70 protein in each time point normalized to the expression levels of the loading control G3BP. The intensity of the signal in each lane was measured using ImageQuant software. Each bar graph represents the ratio of Hsp70 over G3BP for each time point.  The histogram presents the results from (A) as a mean +/- SEM (error bars), from three independent experiments.

In summary, delineating the functional relevance of SGs in senescent cells exposed to a variety of extracellular drugs could be relevant to the treatment of age-related diseases such as cancer. Indeed, many chemotherapeutic agents are used due to their ability to trigger senescence in cancer cells. Though a growing number of small molecules that induce irreversible cell cycle arrest in malignant cells have been recently developed, improvement of cancer treatment is limited, underscoring the need for identifying the mechanisms by which these treatments could modify the behavior of senescent cells [10,20,36-38]. In fact, in some cases, malignant cells exposed to repeated treatments with these molecules may  become promoter of tumorigenesis; possibly by activating the senescence-associated secretory phenotypes (SASPs) [39,40]. Indeed, in the work describing the impact of SASPs on cancer development, the Campisi laboratory showed that while treatment of human prostatic tumor cells with the DNA-damaging agent mitoxantrone (MIT) promote their entry to a senescence state, it also activates SASPs leading to the secretion of promalignant factors such as IL-6 and IL-8 [39]. These two proinflammatory cytokines, are secreted in the microenvironement of senescent cells triggering epithelial-mesenchyme transition and invasiveness, two clear signs of tumor growth and metastasis. Although it is not known whether induction of senescence in the tumor cell itself could enhance their malignant potential at later stages, the activation of the SASPs clearly indicates that these cells facilitate the transformation of neighboring cells that are not tumorigenic to become such. Since chemotherapeutic agents normally cause severe stresses, it is possible that some of them trigger the assembly and disassembly of SGs. If the mRNAs encoding SASPs factors are also recruited to these entities, the repeated cycles of translation inhibition/recovery could over time alter their expression pattern causing their massive synthesis and secretion in the surrounding environment. Exploring this possibility and defining whether or not SG assembly/disassembly plays a role in this outcome could help better understand why after being effective at early stages of the treatment some drugs revert and become promoter of malignancy.

**Figure 2.**
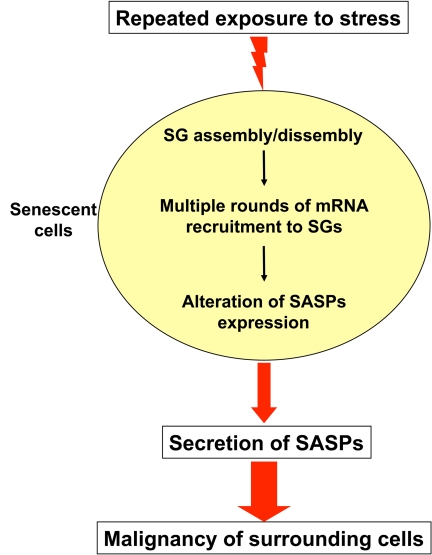
Working model of how repeated exposure to stress could change the pattern of mRNA expression in senescent cells. In response to stresses such as oxidative stress and heat shock senescence cells form a high number of stress granules (SGs). These SGs recruit several mRNAs leading to their translation inhibition. During its lifespan, a living organism is exposed repeatedly to a variety of stresses. This could trigger multiple cycles of SGs assembly/disassembly which in turn change the expression pattern of mRNAs encoding factors responsible of activation the senescence-associated secretory phenotypes (SASPs). Consequently, this could enhance the levels of these SASPs factors that will be secreted in the microenvironment promoting malignancy in neighboring cells.

## References

[1] Campisi J (2007). Aging and cancer cell biology. Aging Cell.

[2] Schmitt CA (2007). Cellular senescence and cancer treatment. Biochim Biophys Acta.

[3] Hayflick L (1965). The Limited in Vitro Lifetime of Human Diploid Cell Strains. Exp Cell Res.

[4] Hayflick L (1998). How and why we age. Exp Gerontol.

[5] Hayflick L (1985). The cell biology of aging. Clin Geriatr. Med.

[6] Shamblott MJ, Axelman J, Littlefield JW, Blumenthal PD, Huggins GR, Cui Y, Cheng L, Gearhart JD (2001). Human embryonic germ cell derivatives express a broad range of developmentally distinct markers and proliferate extensively in vitro. Proc Natl Acad Sci U S A.

[7] Campisi J (2005). Senescent cells, tumor suppression, and organismal aging: good citizens, bad neighbors. Cell.

[8] Campisi J (2005). Aging, tumor suppression and cancer: high wire-act! Mech Agei. g Dev.

[9] Kim Sh SH, Kaminker P, Campisi J (2002). Telomeres, aging and cancer: in search of a happy ending. Oncogene.

[10] Dimri GP (2005). What has senescence got to do with cancer. Cancer Cell.

[11] Beausejour CM, Krtolica A, Galimi F, Narita M, Lowe SW, Yaswen P, Campisi J (2003). Reversal of human cellular senescence: roles of the p53 and p16 pathways. EMBO J.

[12] Krtolica A, Campisi J (2002). Cancer and aging: a model for the cancer promoting effects of the aging stroma. Int J Biochem. Cell Biol.

[13] Krtolica A, Campisi J (2003). Integrating epithelial cancer, aging stroma and cellular senescence. Adv Gerontol.

[14] Parrinello S, Coppe JP, Krtolica A, Campisi J (2005). Stromal-epithelial interactions in aging and cancer: senescent fibroblasts alter epithelial cell differentiation. J Cell Sci.

[15] Brown JP, Wei W, Sedivy JM (1997). Bypass of senescence after disruption of p21CIP1/WAF1 gene in normal diploid human fibroblasts. Science.

[16] Campisi J, Yaswen P (2009). Aging and cancer cell biology, 2009. Aging Cell.

[17] Campisi J (2001). Cellular senescence as a tumor-suppressor mechanism. Trends Cell Biol.

[18] Campisi J (2002). Cancer and aging: yin, yang, and p53. Sci Aging Knowledge Environ.

[19] Campisi J (2003). Cancer and ageing: rival demons. Nat Rev Cancer.

[20] Campisi J, d'Adda di Fagagna F (2007). Cellular senescence: when bad things happen to good cells. Nat Rev Mol Cell Biol.

[21] Lian XJ, Gallouzi IE (2009). Oxidative Stress Increases the Number of Stress Granules in Senescent Cells and Triggers a Rapid Decrease in p21waf1/cip1 Translation. J Biol Chem.

[22] Kedersha NL, Gupta M, Li W, Miller I, Anderson P (1999). RNA-binding proteins TIA-1 and TIAR link the phosphorylation of eIF-2 alpha to the assembly of mammalian stress granules. J Cell Biol.

[23] Anderson P, Kedersha N (2006). RNA granules. J Cell Biol.

[24] von Roretz C, Gallouzi IE (2008). Decoding ARE-mediated decay: is microRNA part of the equation. J Cell Biol.

[25] Bruunsgaard H, Pedersen BK (2000). Special feature for the Olympics: effects of exercise on the immune system: effects of exercise on the immune system in the elderly population. Immunol Cell Biol.

[26] Helenius M, Makelainen L, Salminen A (1999). Attenuation of NF-kappaB signaling response to UVB light during cellular senescence. Exp Cell Res.

[27] Gabai VL, Sherman MY (2002). Invited review: Interplay between molecular chaperones and signaling pathways in survival of heat shock. J Appl Physiol.

[28] Kregel KC, Moseley PL, Skidmore R, Gutierrez JA, Guerriero V Jr (1995). HSP70 accumulation in tissues of heat-stressed rats is blunted with advancing age. J Appl Physiol.

[29] Luce MC, Cristofalo VJ (1992). Reduction in heat shock gene expression correlates with increased thermosensitivity in senescent human fibroblasts. Exp Cell Res.

[30] Effros RB, Zhu X, Walford RL (1994). Stress response of senescent T lymphocytes: reduced hsp70 is independent of the proliferative block. J Gerontol.

[31] Mazroui R, Di Marco S, Kaufman RJ, Gallouzi IE (2007). Inhibition of the ubiquitin-proteasome system induces stress granule formation. Mol Biol Cell.

[32] Anderson P, Kedersha N (2008). Stress granules: the Tao of RNA triage. Trends Biochem. Sci.

[33] Kedersha N, Anderson P (2002). Stress granules: sites of mRNA triage that regulate mRNA stability and translatability. Biochem Soc Trans.

[34] Mazroui R, Sukarieh R, Bordeleau ME, Kaufman RJ, Northcote P, Tanaka J, Gallouzi I, Pelletier J (2006). Inhibition of ribosome recruitment induces stress granule formation independently of eukaryotic initiation factor 2alpha phosphorylation. Mol Biol Cell.

[35] Dang Y, Kedersha N, Low WK, Romo D, Gorospe M, Kaufman R, Anderson P, Liu JO (2006). Eukaryotic initiation factor 2alpha-independent pathway of stress granule induction by the natural product pateamine A. J Biol Chem.

[36] Marusyk A, DeGregori J (2007). Replicational stress selects for p53 mutation. Cell Cycle.

[37] Marusyk A, Wheeler LJ, Mathews CK, DeGregori J (2007). p53 mediates senescence-like arrest induced by chronic replicational stress. Mol Cell Biol.

[38] Collado M, Blasco MA, Serrano M (2007). Cellular senescence in cancer and aging. Cell.

[39] Coppe JP, Patil CK, Rodier F, Sun Y, Munoz DP, Goldstein J, Nelson PS, Desprez PY, Campisi J (2008). Senescence-associated secretory phenotypes reveal cell-nonautonomous functions of oncogenic RAS and the p53 tumor suppressor. PLoS Biol.

[40] Rodier F, Coppe JP, Patil CK, Hoeijmakers WA, Munoz DP, Raza SR, Freund A, Campeau E, Davalos AR, Campisi J (2009). Persistent DNA damage signalling triggers senescence-associated inflammatory cytokine secretion. Nat Cell Biol.

